# High frequency hearing loss may act as a screening index evaluating otolith function in vertigo patients with normal semi-circular canal function

**DOI:** 10.3389/fneur.2022.978490

**Published:** 2022-08-19

**Authors:** Liang Tian, Zhongchun Chen, Jinyu Wang, Lei Zhang, Hao Zhao, Fanglu Chi, Jing Wang

**Affiliations:** ^1^Ear, Nose, Throat (ENT) Institute and Department of Otorhinolaryngology, Eye and ENT Hospital, Fudan University, Shanghai, China; ^2^National Health Council Key Laboratory of Hearing Medicine (Fudan University), Shanghai, China; ^3^Department of Otorhinolaryngology-Head and Neck Surgery, Huashan Hospital, Fudan University, Shanghai, China; ^4^Department Nuclear Medicine, The Affiliated Tai'an City Central Hospital of Qingdao University, Tai'an, China

**Keywords:** high-frequency hearing loss, otolith dysfunction, vertigo, pure tone audiometry, otolith organ function damage

## Abstract

**Objective:**

To investigate whether otolith dysfunction is related to hearing impairment in vertigo patients with normal semicircular canal function, and to clarify the types of hearing impairment that may be related to otolith organ damage.

**Methods:**

The demographic data, pure tone threshold audiometry (PTA) results (air-conduction), data of bithermal and video-head impulse test (vHIT), and vestibular evoked myogenic potential (VEMP) results (reaction threshold, P1–N1 amplitude) of patients with vertigo in outpatient clinic from April 2017 to January 2020 were collected. The clinical records of 51 vertigo patients with normal semicircular canal function were included in this study. Low-frequency, speech-frequency, high-frequency, full-frequency PTA were defined as the average of PTA in different frequency bands, respectively (low: 0.125, 0.25, 0.5 kHz; speech: 0.5, 1, 2 kHz, high: 4, 8 kHz, full 0.125–8 kHz). The correlations between hearing impairment in different frequency bands and otolith function impairment were analyzed.

**Results:**

The mean thresholds of 51 patients (102 ears) in low-PTA, speech-PTA, high-PTA, full-PTA were 20.95 ± 6.01, 21.92 ± 6.90, 40.12 ± 17.47, 26.97 ± 8.53 dB nHL, respectively. Among 102 ears, 87 ears (85.3%) could elicit c-VEMP waveforms and 65 ears (63.7%) had o-VEMP waveforms. The mean threshold and P1–N1 amplitude of c-VEMP were 83.10 ± 6.96 dB nHL and 176.79 ± 103.10 uV, while those of o-VEMP were 87.92 ± 5.99 dB nHL and 21.45 ± 32.22 uV. The mean threshold in high-PTA was significantly linearly correlated with c-VEMP threshold (*P* = 0.01) and P1–N1 amplitude (*P* = 0.028). There were not significant linear correlations between the mean threshold in each frequency band of PTA and o-VEMP threshold (low-PTA: *P* = 0.266, speech-PTA: *P* = 0.33, high-PTA: *P* = 0.311) or P1–N1 amplitude (low-PTA: *P* = 0.414, speech-PTA: *P* = 0.069, high-PTA: *P* = 0.08).

**Conclusions:**

There is a positive linear correlation between saccule dysfunction and high-frequency hearing impairment in vertigo patients with normal semi-circular canal function. High frequency hearing loss can be expected in patients who have saccular damage. It suggests that high frequency hearing loss in PTA may act as a screening index that otolith organ function should be comprehensively evaluated.

## Introduction

Vertigo is one of the most common symptoms of nervous system diseases, which is often encountered in neurology, otolaryngology, and general medical practice (1). Vertigo has a 12-month prevalence of 5% and leads to falls, especially in older patients, of which 23% suffer from fall-associated trauma at least once a year (2). Although most vertigo diseases are not life-threatening, it obviously affects people's normal life and social activities, and also leads to anxiety and depression.

Three semicircular canals (SCCs) and otolith organs containing saccule and utricule constitute the terminal organs of vestibular system. Vertigo is mainly considered to be caused by the dysfunction of SCC sensing the stimulation of rotational acceleration, and the function of SCCs is usually evaluated by Bithermal and video-head impulse tests (vHIT) (3). In clinic, patients only with abnormal otolith organ function but with normal SCCs function could be found, which is called isolated otolith dysfunction (iOD) (4). Vestibular symptoms caused by iOD have traditionally been suggested as a tilting sensation, a sense of moving, or feelings of falling (5). Furthermore, swaying, a pulling sensation in the anteroposterior direction, and a somatosensory illusion of walking on pillows or on uneven ground were suggested to originate from iOD (6). Recurrent drop attacks also represent a well-known symptom characteristic of iOD (7). However, patients with IOD often have vertigo symptoms, which is not uncommon in clinic, and a part of iOD patients with recurrent rotatory vertigo may progress into Meniere's Disease in the future (4), which means that otolith organ damage is likely to be the early stage of some vertigo diseases. Therefore, it will be of positive significance for the prevention of disease progression if otolith organ dysfunction can be screened out early.

On account of the close physical connection between cochlea and otolith organs, they are frequently affected by the same factors (8, 9). It is very likely that they may have certain correlation on function. In this study, vertigo patients with normal semicircular canal function were enrolled and the relationship between the mean hearing thresholds of PTA in different frequency bands and VEMP test results were analyzed and compared in order to discuss whether there was a correlation between the functional status of cochlea and that of otolith organs.

## Materials and methods

### Study design and subjects enrolled

We collected and reviewed the medical records of 235 consecutive vertigo patients with detail evaluation results of VEMPs testing to air-conducted sound (ACS VEMP), caloric test and vHIT test between April 2017 and January 2020 at the outpatient clinic of the Eye, Ear, Nose, and Throat Hospital affiliated with Fudan University. Fifty-one cases met the following inclusion criteria were included in this study: (a) without abnormal SCC function evaluated by bithermal test and vHIT test; (b) no obvious sudden hearing loss which could be self-perceived; (c) with detail results of PTA test and VEMP test. Exclusion criteria: (1) combined/previous history of sudden hearing loss, head trauma, ear surgery, psychological diseases and mental diseases; (2) significant air-bone gap in PTA; (3) the other obvious health problems that could affect the test results (such as gravis myasthenia).

The study was approved by the research ethics committee of the Eye, Ear, Nose, and Throat Hospital affiliated with Fudan University (No. 2017025-1). All patients provided signed informed consent.

### Audiometry

The mean threshold values at different frequency bands were calculated as follow: low-frequency PTA (low-PTA) was gained by averaging air conduction threshold at 0.125, 0.25, 0.5 kHz; speech-frequency PTA (speech-PTA) was the average of threshold values at 0.5, 1, and 2 kHz; high-frequency PTA (high-PTA) was that at 4 and 8 kHz; full-frequency PTA (full-PTA) was the average of threshold values from 0.125 to 8 kHz. The classification of hearing loss refers to the standard issued by WHO in 2021: normal hearing <20 dB, mild loss 20 to <35 dB, moderate loss 35 to <50 dB, moderately severe loss 50 to <65 dB, severe loss 65 to <80 dB, profound 80 to <95 dB, complete or total loss 95 dB or greater.

### Vestibular-evoked myogenic potential test

The cervical and ocular VEMPs test were performed on patients in a supine position in a sound-proof room. Using air-conducted sound (ACS), a total of 120 auditory stimuli (short tone burst, 500 Hz, 2-ms rise/fall time, 2 ms plateau time) were applied to each ear *via* calibrated insert headphones. The electromyography signals were amplified in a Bio-Logic Navigator PRO system (Bio-logic Auditory Evoked Potential Ver. 7.0.0 software, Bio-Logic Systems Corp, Mundelein, IL). Exact location and detailed method of electrode placement in the test of VEMPs have been described in our previous study (9, 10).

The starting stimulus intensity was 95 dB nHL to confirm a VEMP response, then decreased in 5 dB nHL per step until the VEMP responses were not detected. Response thresholds were defined as the minimum stimulus intensities of the characteristic waveform. The elicitation of cVEMP was confirmed when the characteristic P13–N23 (positive-negative waveform) appeared. An absence of cVEMP was determined when the typical waveforms could not be elicited or were unrepeatable. The elicitation of oVEMP was defined as the occurrence of the characteristic N10–P15 (negative-positive waveform). An absence of oVEMP was established when the typical waveforms could not be elicited or were unrepeatable. The amplitudes of cVEMP and oVEMP were recorded as the voltage difference between the first and second peaks of the response, that is, P13–N23 for cVEMP, and N10–P15 for oVEMP (11).

### Statistical analysis

Data were analyzed using IBM SPSS Statistics 25. All continuous data were presented as the mean ± standard deviation (SD). The mean values were compared by independent sample *t*-test and 1-way ANOVA. The diagnostic accuracy of PTAs of different frequency bands in patients with severe otolith organ damage (no cVEMP/oVEMP response) were evaluated using receiver operating characteristic (ROC) curve analysis. The correlations between PTAs of different frequency bands and VEMP results were analyzed by linear regression. Statistical significance was set at *p* < 0.05.

## Results

### Demographic characteristics

**Figure 1:**
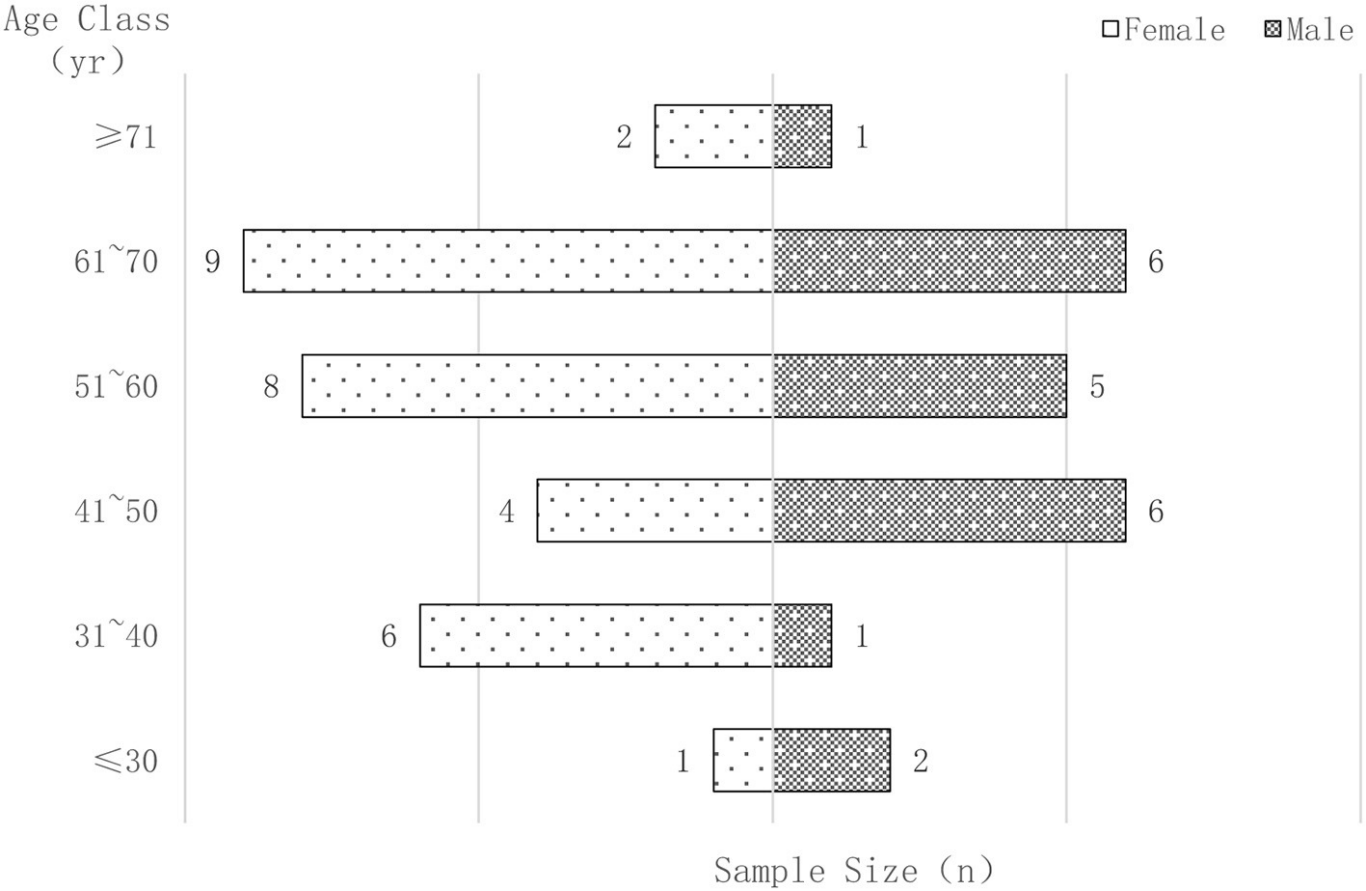
The gender-age distribution of all samples. Numbers outside bars indicate sample size.

Fifty-one patients who met the criteria were enrolled in this study, including 30 females and 21 males. Their ages ranged from 15 to 81 years, with a mean age of 53.06 ± 13.71 years. The majority (88.24%) of the subjects were between 30 and 70 years old. The gender and age characteristics of all patients are shown in Figure 1.

### Clinical characteristics in PTA results

**Figure 2:**
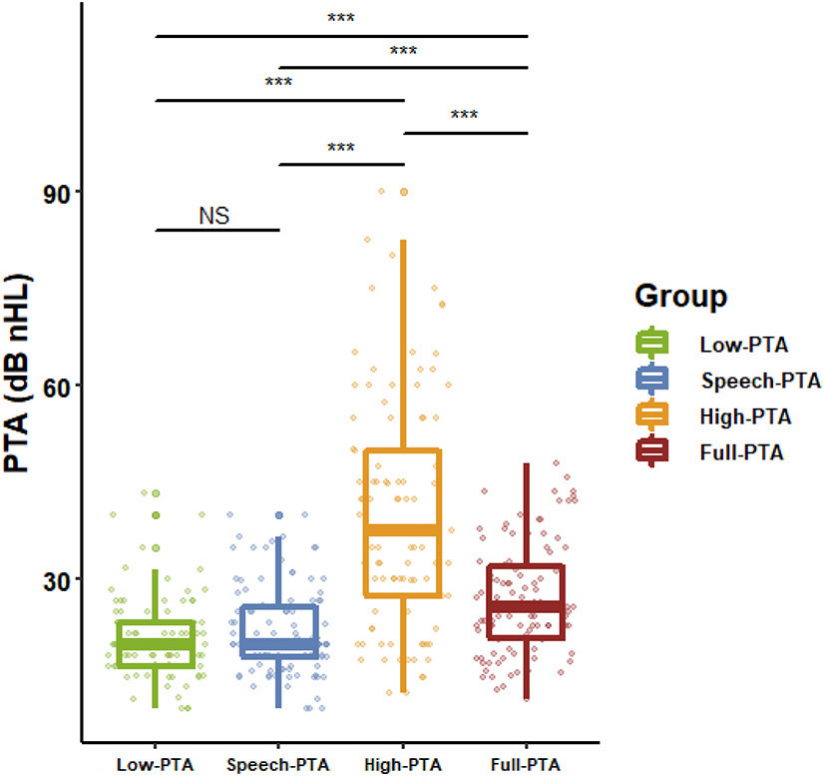
The distribution of hearing thresholds at various frequency bands. Except for low frequency and speech frequency, the average values of PTA at all other frequency bands were significantly different (*n* = 102, *p* < 0.001, 1-way ANOVA and Dunnet's posttest). ***p < 0.001. NS: *p* > 0.05.

According to the WHO classification standard for hearing loss in 2021, 21 cases (41.18%) had normal hearing, 27 (52.94%) had mild hearing loss and 3 (5.88%) had moderate hearing loss. A total of 58 (56.86%) ears appeared abnormal hearing threshold in low frequency band, 61 (59.80%) ears in speech band, 92 (90.20%) ears in high frequency band and 79 (77.45%) ears in full frequency band. The majority of patients have normal or slight decrease in low-frequency and speech frequency hearing, and the proportion and damage degree of high frequency hearing loss are relatively higher than those of low-frequency and speech frequency hearing loss. The hearing thresholds at different frequency bands of 102 ears are shown in Figure 2.

### Clinical characteristics in VEMP results

In all 102 ears, 87 ears (85.29%) could elicit cVEMP and 65 ears (63.72%) could elicit oVEMP. The mean threshold and the average P1–N1 amplitude of c-VEMP were 83.10 ± 6.96 dB nHL and 176.79 ± 103.10 uV, and those of o-VEMP were 87.92 ± 5.99 dB nHL and 21.45 ± 32.22 uV. The detail results of cVEMP and oVEMP were listed in Table 1.

**Table 1:** cVEMP and oVEMP results of all enrolled patients.

	**cVEMP**	**oVEMP**
Response/No response (ears)	87 (85.29%)/15 (14.71%)	65 (63.72%)/37 (36.28%)
Bilateral response (cases/proportion)	39 (76.74%)	26/50.98%
Unilateral response (cases/proportion)	9/17.65%	13/25.49%
No response in both ears (cases/proportion)	3/5.88%	12/23.53%
Total (cases/proportion)	51/100%
Threshold (mean ± SD, dB nHL)	83.10 ± 6.96	87.92 ± 5.99
P1–N1 amplitude (mean ± SD, uV)	176.79 ± 103.10	21.54 ± 32.22

### Comparison of PTA results between VEMP evoked and non-evoked groups

**Figure 3:**
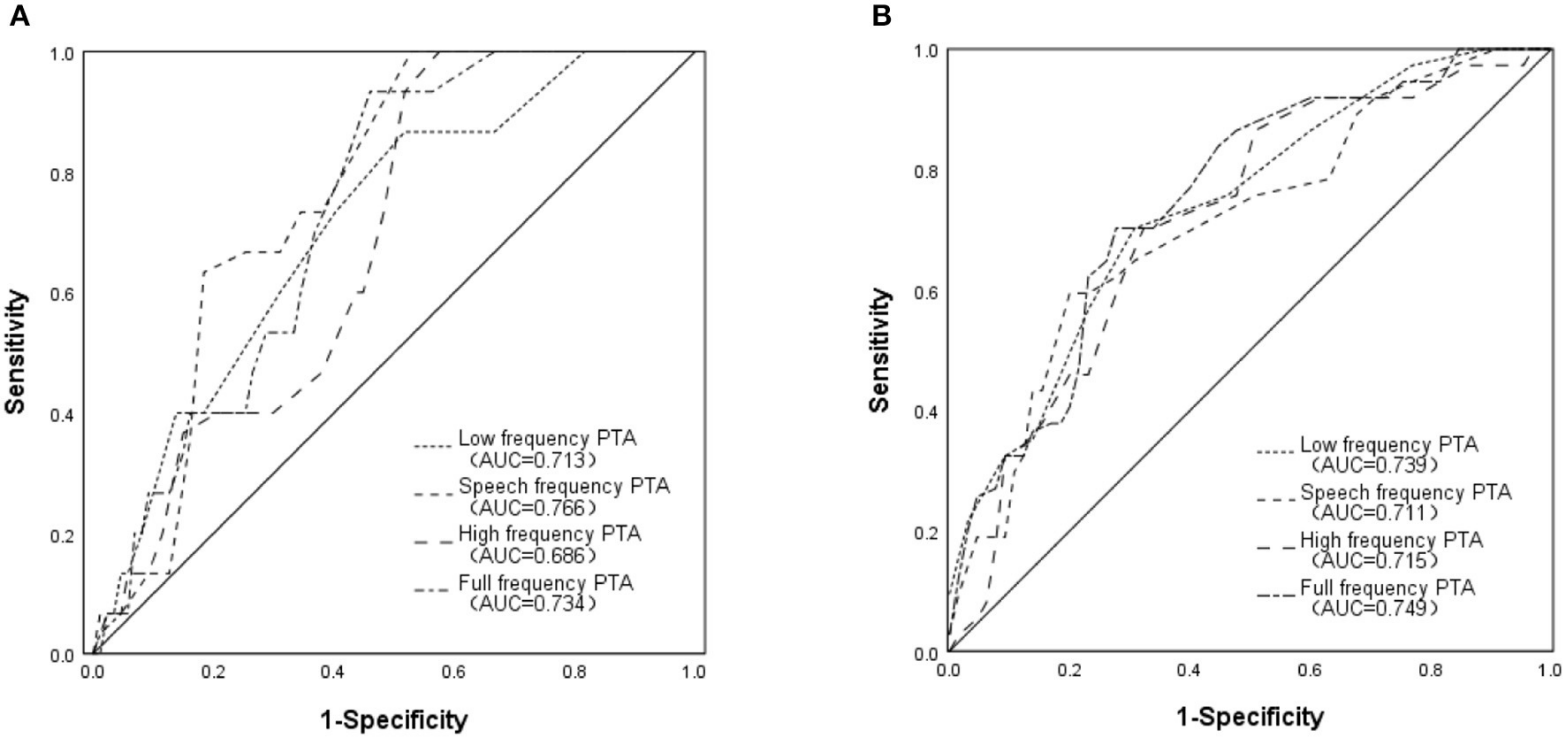
Receiver operating characteristic curves of PTAs at different frequency bands in predicting ipsilateral otolith organ dysfunction **(A)** Evaluating saccular function by predicting whether cVEMP waveforms could be elicited; **(B)** Evaluating utricular function by predicting whether oVEMP waveforms could be elicited.

All 102 ears were divided into evoked group and non-evoked group according to whether the waveforms of cVEMP could be recorded. There was significant difference between these two groups by comparing the mean hearing threshold values of low frequency, speech frequency, high frequency and full frequency. The same results were also found in the groups divided by the oVEMP results (Table 2). ROC analysis showed the certain diagnostic accuracy of mean hearing threshold values at low frequency, speech frequency, high frequency and full frequency in estimating whether VEMP waveforms could be evoked (Figure 3).

**Table 2:** Comparison of PTAs between VEMP evoked and non-evoked groups.

	**cVEMP**			**oVEMP**		
	**Evoked**	**Non-evoked**	***P*-value**	**Evoked**	**Non-evoked**	***P*-value**
Low-PTA (dB nHL)	20.34 ± 5.84	24.45 ± 6.00	0.024*	19.00 ± 4.48	24.37 ± 6.85	<0.001**
Speech-PTA (dB nHL)	21.10 ± 6.75	26.69 ± 5.85	0.003**	20.00 ± 5.97	25.30 ± 7.18	<0.001**
High-PTA (dB nHL)	38.37 ± 17.45	48.87 ± 15.52	0.031*	35.58 ± 16.87	47.51 ± 16.17	0.001**
Full-PTA (dB nHL)	26.45 ± 8.87	33.07 ± 7.62	0.008**	24.71 ± 7.94	32.19 ± 8.78	<0.001**

*p < 0.05, **p < 0.01.

### Correlation analysis between PTA and VEMP results

In 87 ears which could induce c-VEMP, there was statistically significant correlation between the mean threshold in full-frequency PTA and c-VEMP threshold, so did P1–N1 amplitude of c-VEMP. Further analysis found that high-PTA was significantly linearly correlated with c-VEMP threshold and P1–N1 amplitude, while the statistically significant correlations were not found in low-PTA, speech-PTA compared with c-VEMP threshold or P1–N1 amplitude. PTAs of different frequency ranges were not significantly linearly correlated with o-VEMP threshold or P1–N1 amplitude. Correlation analysis between PTAs at different frequency bands and VEMP results were listed in Table 3.

**Table 3:** Correlation between PTAs at different frequency bands and VEMP results by linear regression.

	**cVEMP**				**oVEMP**			
	**Threshold**		**Amplitude**		**Threshold**		**Amplitude**	
	***F* value**	***P*-value**	***F*-value**	***P*-value**	***F*-value**	***P*-value**	***F*-value**	***P*-value**
Low-frequency PTA	0.835	0.406	1.263	0.206	1.258	0.266	0.676	0.414
Speech-frequency PTA	2.755	0.101	3.157	0.079	0.963	0.330	3.416	0.069
High-frequency PTA	7.037	0.010**	4.967	0.028*	1.043	0.311	3.170	0.080
Full-frequency PTA	5.298	0.024*	4.833	0.031*	1.379	0.245	3.424	0.069

*p < 0.05, **p < 0.01.

## Discussion

Otolith organs play an important role in maintaining balance, orientation and cognitive function (12–14). Patients with otolith organs disorder often appear the sense of tilt, instability or somatosensory illusion (15, 16). Hence, people usually pay attention to the damage of semicircular canal which mainly manifest vertigo and the functional state of otolith organs is easy to be ignored. However, clinically, it is found that the dysfunction of otolith organs may be the early manifestation of damage to peripheral vestibular organs (17). With the improvement of vestibular system detection technology and rising demand for high quality of life, more and more people begin to pay attention to the functional evaluation and protection of otolith organs.

VEMP, a common method to evaluate otolith organs, needs high technique requirement, takes a long test time, and is relatively expensive, which limits the wide application of this technique in clinic. How to simply and quickly screen out patients with otolith organ dysfunction or potential damage and intervene in time is a problem worthy of discussion. The cochlea and saccule are adjacent in anatomy and homologous in the development of the inner ear, which suggesting that they may have some correlation in function. In previous studies, we found that patients with severe sensorineural deafness were often accompanied by otolith organ dysfunction (18–22).

In this study, vertigo patients with normal semicircular canal function were enrolled. It was found that otolith organ dysfunction and hearing loss detected by VEMP and PTA in some patients, which means that vertigo patients with normal semicircular canal function do have the state of impaired cochlea and otolith organ function. By comparing the mean hearing threshold values of low frequency, speech frequency, high frequency and full frequency in patients with evoked and non-evoked VEMP normal waveform, it was found that there were significant differences between these two groups, which suggested that auditory function of each frequency band may have abnormal performance in vertigo patients with severe otolith organ damage. ROC analysis also showed that it had certain diagnostic significance for the hint of severe otolith organ damage by assessing full band auditory function.

cVEMP and oVEMP can reflect the function of saccule and utricule, respectively. Further analysis was made about the relationship between hearing threshold values of each frequency band in PTA and the results of cVEMP/oVEMP, respectively. It was found that there was a significant linear correlation between hearing threshold in high frequency band and cVEMP results, including reaction threshold and P1–N1 amplitude. The more obvious the high-frequency hearing loss was, the more seriously the saccule function was damaged, which suggested that there was a consistency between the function state of cochlear hair cells in the basal turn of cochlea that can feel high-frequency sound stimulation and the functional state of saccule. Cochlea and Saccule are anatomically adjacent. Meanwhile, they are homologous and developed from the cochlear sac (23, 24). Those are the possible reasons for the close correlation between their functions. Hair cells located in basal turn of cochlear are sensitive to the change of blood supply (25). High-frequency hearing loss often indicates that blood supply in basal turn of cochlea is insufficient. The main supply vessel of saccule and cochlear basilar turn is vestibular cochlear artery (26). Therefore, if the artery is involved, the hair cells in these two functional areas may be damaged at the same time. However, if the damage is not caused by vessel factors but the others, such as infection, noise, immune diseases, trauma, the conclusion that there was relationship between these two parts may not be reached. Low frequency hearing loss is mostly related to labyrinthine hydrops of inner ear, which is caused by many factors. Therefore, it is hard to find the certain relationship between the simple low-frequency hearing loss and the function of otolith organs. As mentioned above, the relationship of cochlear basal turn and saccule in anatomical location, development and supply vessels confirms the possibility of the findings in this study. There was no significant correlation between the results of oVEMP and PTA. The reasons may be as follows: Firstly, there is a relative long distance between cochlea and utricule. Secondly, cochlea is developed from cochlear sac, while utricule from vestibular sac. Thirdly, cochlea and utricule have different blood supply arteries, the former is vestibular cochlear artery, and the latter is anterior vestibular artery (27). In addition, the signal of cVEMP is easier to record and more stable than that of oVEMP. It is another reason why cVEMP is easy to find meaningful results.

VEMP is a relatively expensive test. It takes a long time to fulfill the examination and requires patients to cooperate perfectly in order to obtain accurate results. Therefore, it could not be widely used in the function screening of otolith organs in clinic. This study found that the results of PTA in high-frequency band are linear correlation to the results of cVEMP. Patients with high-frequency hearing loss are easy to be combined with the increase of cVEMP response threshold and the decrease of P1–N1 amplitude. Therefore, PTA may be used as a basic test for otolith organ function screening, and the mean hearing threshold in high-frequency area may be used as a predictor to indicate whether saccule function probably decline, so as to screen out the patients with otolith organ function impairment in the early stage, or monitor the disease progression for those patients who have potential problem and probably develop into otolith organ dysfunction.

## Conclusion

This study confirmed that there is a linear correlation between the hearing threshold in high frequency band of PTA and cVEMP results, including the threshold and P1–N1 amplitude. It suggested that there is a correlation between cochlea and saccule function. High frequency hearing loss can be expected in patients who have saccular damage. Hence, PTA may be used as a basic test for otolith organ function screening, and high frequency hearing loss may be used as an index to comprehensively evaluate otolith organ function in vertigo patients with normal semicircular canals function.

## Data availability statement

The original contributions presented in the study are included in the article/supplementary material, further inquiries can be directed to the corresponding author/s.

## Ethics statement

The studies involving human participants were reviewed and approved by Ethics Committee of the Eye, Ear, Nose, and Throat Hospital affiliated with Fudan University (No. 2017025-1). The patients/participants provided their written informed consent to participate in this study.

## Author contributions

JingW designed the study and reviewed the manuscript. LT, ZC, JinyW, and LZ collected and analyzed the data and drafted the manuscript. HZ and FC collected the data and performed the statistical analysis. All authors were responsible for the final approval of the version to be published.

## Funding

This study was supported by the General Program of National Natural Science Foundation of China (NSFC 81870724), the Innovation Project of Shanghai Municipal Science and Technology Commission (19441900400), and the Shanghai Municipal Health Commission (201740018).

## Conflict of interest

The authors declare that the research was conducted in the absence of any commercial or financial relationships that could be construed as a potential conflict of interest.

## Publisher's note

All claims expressed in this article are solely those of the authors and do not necessarily represent those of their affiliated organizations, or those of the publisher, the editors and the reviewers. Any product that may be evaluated in this article, or claim that may be made by its manufacturer, is not guaranteed or endorsed by the publisher.
